# Insulin Glargine Biosimilar Prescribing and Cost Trends in the United Kingdom’s Primary Care from 2020 to 2024

**DOI:** 10.3390/pharmacy13030085

**Published:** 2025-06-14

**Authors:** Murtada Alsaif, Zoë Blumer

**Affiliations:** 1 PharmaSaif Ltd., Slough SL2 2LR, UK; 2 Zoe Blumer Consulting Ltd., Bognor Regis PO21 3TL, UK

**Keywords:** insulin, insulin glargine, biosimilar, primary care, prescribing data, real-world data, diabetes

## Abstract

Background/Objectives: Long-acting insulin glargine (iGlar) has been available as a biosimilar since 2014 in the UK. We reviewed previous prescribing to evaluate if the anticipated cost savings with biosimilars were realized with iGlar. Methods: This study investigated prescribing patterns of long-acting iGlar (100 units/mL) in cartridges and pre-filled pens from 2020 to 2024 across primary care organizations in England, Northern Ireland, Scotland, and Wales. Results: iGlar prescribing declined in all of the four nations. From 2020 to 2024, the total prescribed quantity of biosimilars persistently increased in all countries, reaching 24% in England, 5% in Northern Ireland, 24% in Scotland, and 11% in Wales, all in 2024. Consequently, the proportion of Lantus prescriptions (as quantity) decreased but continued to exceed that of all available iGlar products combined in all countries in all years analyzed. By 2024, Lantus was also priced lower than the most common biosimilar, Abasaglar, across all nations. Conclusions: The introduction of biosimilars does not automatically result in altered prescribing practices, though we show that the most commonly prescribed iGlar was also the least expensive product at the end of the analysis period. At launch and for several years after, biosimilars failed to gain strong utilization, despite cost advantages, highlighting the need for active switching policies and prescriber engagement.

## 1. Introduction

A biosimilar is a biological medicinal product that is highly similar to an already authorized biological medicine, known as the reference product (RP, or originator product), in terms of quality, safety, and efficacy [1]. RPs are often expensive [2], representing approximately one-third of total pharmaceutical spending for health systems [3]. Biosimilars are thought of as affordable alternatives, through price competition or mandatory discounts [2,3].

In November 2022 the Medicines and Healthcare products Regulatory Agency (MHRA) released updated Guidance on the licensing of biosimilar products in the United Kingdom (UK), stating that biosimilars and the RP are interchangeable [4]. Interchangeability within clinical practice offers benefits in terms of improved access to treatment, sustainability of healthcare resources, contribution to better delivery of patient care [5], and reduced pharmaceutical spending for payers [5,6].

In conflict with the MHRA, the British National Formulary (BNF) advises prescribers: “Biological medicines must be prescribed by brand name and the brand name specified on the prescription should be dispensed in order to avoid inadvertent switching. Automatic substitution of brands at the point of dispensing is not appropriate for biological medicines” [7]. As such, switching brands must be initiated by the prescriber and should not be performed by the dispenser [4].

Long-acting insulin glargine (iGlar) biosimilars have been available in the UK since 2014. The indication of iGlar biosimilars is extrapolated from Lantus, which is the treatment of diabetes mellitus in adults, adolescents, and children aged 2 years and above [8]. Two biosimilars, Abasaglar (previously Abasria) and Semglee, were approved by the European Medicines Agency in September 2014 and January 2018, respectively [9,10]. In the UK, biosimilar adoption is primarily driven by cost-saving priorities, which can be influenced by national procurement agreements, local formulary positioning, and prescriber preference. The primary care organizations (PCOs) included in our analysis were Integrated Care Systems (ICSs) in England, Health and Social Care Trusts (HSTPs) in Northern Ireland, Health Boards (HBs) in Scotland, and HBs in Wales. PCOs provide system-wide prescribing polices and clinical guidance supporting safe, effective and cost-effective prescribing across primary and secondary care.

In 2020, Agirrezabal et al. published the budget impact of the uptake of Abasaglar in primary care in England between 2015 and 2018, inclusive. The analysis demonstrated variation in the uptake across PCOs in England, ranging from 0 to 53.3%. The total percentage of savings realized was reported as 3.42% of the total achievable savings during the period analyzed, demonstrating considerable potential savings were being missed due to the lack of biosimilar uptake [11].

The objective of our analysis was to investigate prescribing and cost trends between 2020 and 2024 in PCOs in England, Northern Ireland, Scotland, and Wales, in light of the substantial unrealized savings identified by Agirrezabal et al. [11] and what seems like contradictory advice from the MHRA [4] and the BNF [7].

## 2. Materials and Methods

### 2.1. Scope and Included Products

We reviewed the Dictionary of Medicines and Devices (dm+d) [12] to identify iGlar products. The dm+d organizes medicines under their active ingredient, referred to as the Virtual Therapeutic Moiety (VTM). Each VTM encompasses Virtual Medicinal Products (VMPs), which are generic descriptions of medical products. In turn, each VMP encompasses Actual Medicinal Products (AMPs), representing specific branded or unbranded medicines that can be found on the pharmacy shelf. Prescriptions can be written according to their VMP (generic prescribing) or AMP (branded prescribing) description. BNF codes, which were used to extract prescribing data, are available for both VMPs and AMPs.

A full list of all insulin glargine products and their coding details can be found in the Supplementary Materials. According to the dm+d, five VMPs contain insulin glargine, of those, only two have more than one AMP (i.e., different products from multiple manufacturers). This means that prescribers have only one choice if they want to prescribe insulin glargine as 100 units/mL in a 10 mL vial or the 300 units/mL variations. However, prescribers can choose between the reference brand and other biosimilars, if they want to prescribe insulin glargine 100 units/mL cartridges or pre-filled disposable devices (PFDs). Therefore, only insulin glargine 100 units/mL cartridges and PFDs were included in this study (Table 1).

### 2.2. Prescribing Data Extraction and Analysis

This analysis included community prescribing data for England [14], Northern Ireland [15], Scotland [16], and Wales [17], which are published monthly at the GP practice level. In line with the previously defined scope, hospital prescribing data were not included in the main analysis as they are not freely available for Wales and Northern Ireland, and different biosimilars are not distinguished in England data, which is an essential requirement of this study. When investigated, we found community prescribing data to be representative of iGlar prescribing in England (hospital prescribing data extracted for the year 2024 and detailed in the Supplementary Materials), and we assume this can be generalized to Northern Ireland, Scotland, and Wales.

All community prescribing data extracts were based on the BNF codes and extraction was performed between January 2025 and March 2025, any amendments applied after this date were not included. AMPs without a BNF code were not included in the analysis as searching the prescribing data by product description may introduce inaccuracies in the final extract.

Prescribing data for England, Northern Ireland, and Wales includes both the net ingredient cost (represents the Drug Tariff price or the list price, as appropriate) and the actual price (considers the national average discount and some payments to dispensers). This study used the actual price reported, as it better represents what the commissioner pays for prescribing. For Scotland, only the gross ingredient cost (representing the Drug Tariff price or the list price) is reported, and that was used in the analysis.

All data were extracted using R version 4.2.2, The R Foundation for Statistical Computing, and further analyzed with Microsoft Excel, Microsoft Office Professional Plus 2021. In R, subsets were extracted based on the BNF code to the PCO level per month. In Excel, prescribed quantity and cost were aggregated to the national or PCO level and presented per month or per year, depending on the analysis. Prescribing data were not transformed or manipulated, beyond aggregating them as described here.

## 3. Results

For a detailed breakdown of prescribing at the country level, please see the Supplementary Materials.

### 3.1. Quantity Prescribed

#### 3.1.1. England

**Figure 1:**
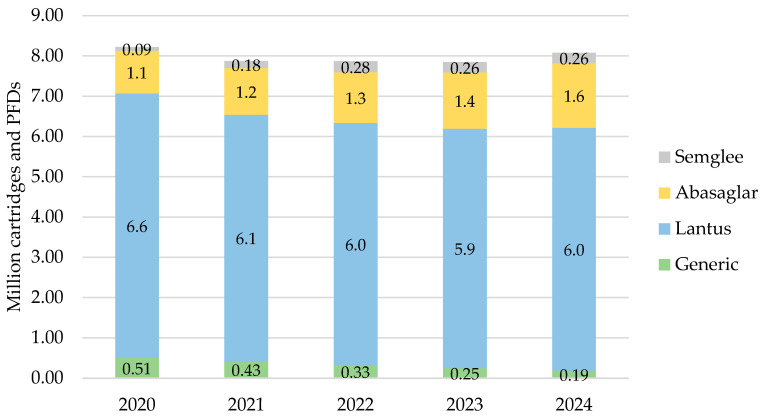
Prescribed quantity of insulin glargine in England, 2020 to 2024. Legend: The y-axis is the sum of prescribed cartridges and prefilled devices (PFDs). The label in each bar segment is the sum of cartridges and PFDs (as prescribed quantity) per brand in millions.

In England, iGlar prescribing was highest in 2020 at a total of 8.2 million cartridges and PFDs, which then declined over 2021, 2022, and 2023 but increased again to 8.1 million in 2024 **(**Figure 1). Lantus pens represented 66% of all prescribed iGlar in 2020 and 65% in 2024.

There was a consistent rise in the proportion of iGlar prescribed as Abasaglar and Semglee. The proportion of biosimilar to RP (ignoring generic prescribing) from 2020 to 2024 was 15%, 18%, 20%, 22%, and 24%. This increase was primarily driven by Abasaglar, where the quantity of prescribing was six times higher than Semglee, on average over 5 years. This was coupled with a stable decline in generically prescribed iGlar of 63% over 5 years (from 510,474 to 189,417 cartridges and PFDs in 2020 to 2024, respectively). The proportion of Lantus pens remained stable between 2020 and 2024.

For all products, prescribed cartridges declined by 33% from 2020 to 2024 (from 1.3 million to 888,910 cartridges), but prescribed PFDs increased by 4% in the same period (from 6.8 million to 7.2 million PFDs). Abasaglar pens were the only product that consistently increased in prescribing over the 5 years.

#### 3.1.2. Northern Ireland

**Figure 2:**
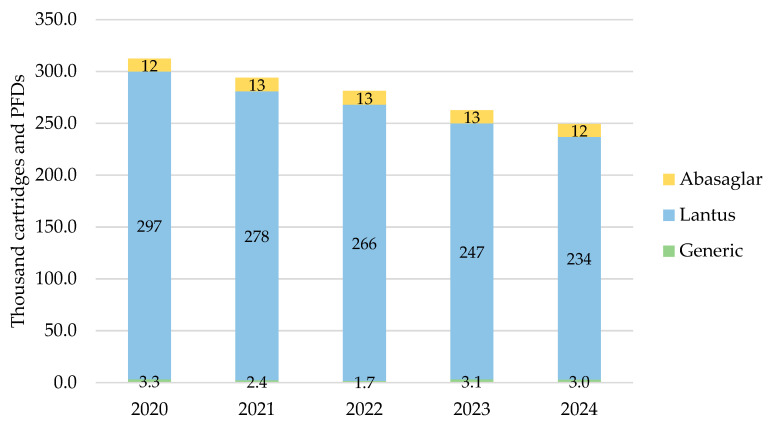
Prescribed quantity of inulin glargine in Northern Ireland, 2020 to 2024. Legend: The y-axis is the sum of prescribed cartridges and prefilled devices (PFDs). The label in each bar segment is the sum of cartridges and PFDs (as prescribed quantity) per brand in thousands.

In Northern Ireland, the total prescribed cartridges and PFDs of iGlar were highest in 2020 at 312,289 items, declining year-on-year to 2024 (Figure 2). Lantus pens represented 90% of all prescribed iGlar over the 5-year period.

The only biosimilar prescribed was Abasaglar, as a proportion of branded prescribing, it comprised 4% in 2020 and 2021, and this increased to 5% in 2022, 2023, and 2024. Generic prescribing was insignificant, ranging from 0.6% to 1.2% of all products in scope over the 5 years.

The number of prescribed pens and PFDs dropped by 20% (2024 vs. 2020) driven by a drop in the number of Lantus pens prescribed.

#### 3.1.3. Scotland

**Figure 3:**
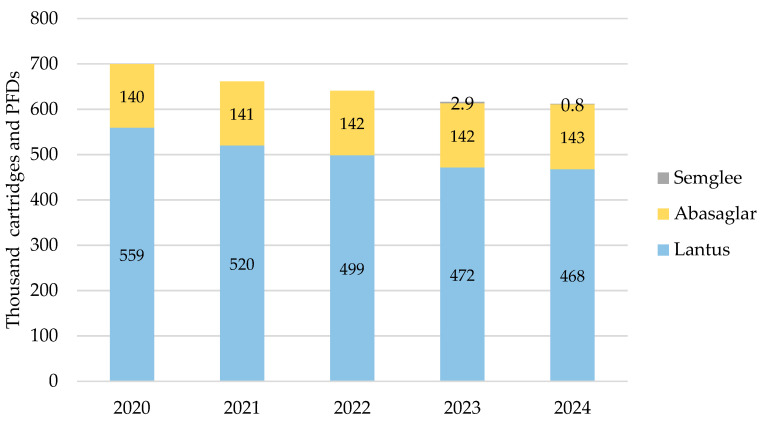
Prescribed quantity of insulin glargine in Scotland, 2020 to 2024. Legend: The y-axis is the sum of prescribed cartridges and prefilled devices (PFDs). The label in each bar segment is the sum of cartridges and PFDs (as prescribed quantity) per brand in thousands.

iGlar prescribing also declined in Scotland over the analysis period. It peaked in 2020 with 699,628 cartridges and PFDs and dropped year-on-year by 13% (2024 vs. 2020) (Figure 3). Again, Lantus was the most commonly prescribed brand at 62% to 63% over 5 years. No generic prescriptions for iGlar were reported in Scotland between 2020 and 2024.

Biosimilar prescribing comprised Abasaglar only for 2020, 2021, and 2022, while the first prescription for Semglee pens was issued in May 2023. Biosimilar prescribing started at 20% in 2020 and rose by 1% year-on-year to 24% in 2024.

#### 3.1.4. Wales

**Figure 4:**
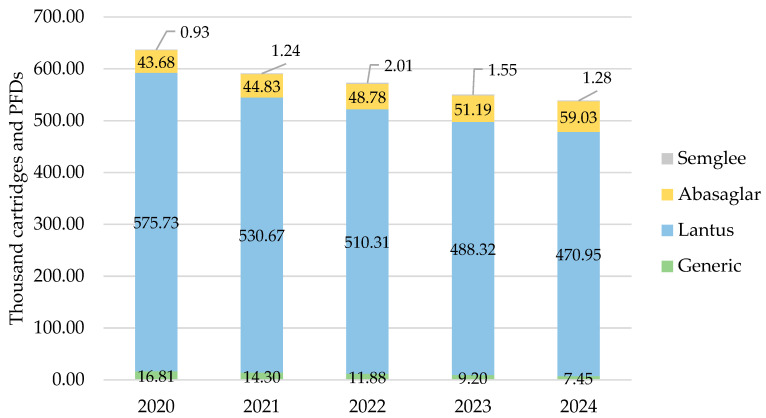
Prescribed quantity of insulin glargine in Wales, 2020 to 2024. Legend: The y-axis is the sum of prescribed cartridges and prefilled devices (PFDs). The label in each bar segment is the sum of cartridges and PFDs (as prescribed quantity) per brand in thousands.

As with all other countries, iGlar prescribing declined in Wales. It peaked in 2020 with 637,137 cartridges and PFDs and dropped year-on-year by 15% (2024 vs. 2020) (Figure 4). Lantus was the most commonly prescribed brand at 80% over 5 years.

Biosimilar prescribing started at 7% in 2020 and rose by 1% year-on-year to 11% in 2024.

### 3.2. Actual Cost of Prescribing

#### 3.2.1. England

**Figure 5:**
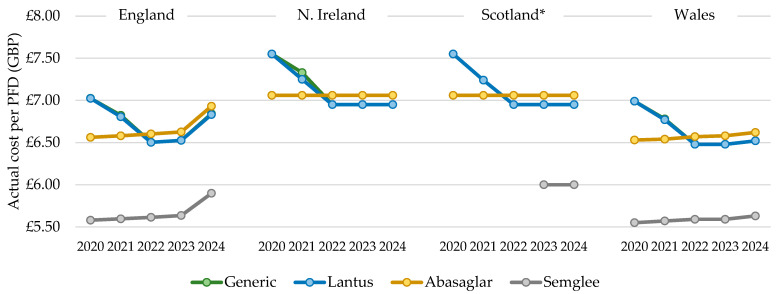
Cost per single pre-filled device (PFD), 2020 to 2024. * Gross ingredient cost per PFD. Legend: The cost per pre-filled device (PFD) was calculated by dividing the total annual cost by the total annual prescribed quantity.

The total actual cost of prescribing mirrored the general patterns described above for quantity; however, the cost per PFD prescribed increased over time with all biosimilars but reduced and then increased for Lantus products. In 2022, the cost per PFD for the dominant biosimilar, Abasaglar, became more expensive than the RP, Lantus, and this trend was maintained in 2023 and 2024 (Figure 5). For all products, the cost/cartridge was within GBP 0.02, relative to their PFD. The cost per generically prescribed iGlar closely matched the RP, Lantus, from 2020 to 2024, indicating that Lantus utilization declined through prescriptions that were written generically. The list price of Lantus pens is GBP 6.95 per PFD [12], and this was implemented in July 2021 and the Abasaglar list price is GBP 7.056 per PFD [12]. The actual price in 2024 for a PFD of Lantus was GBP 6.83 and GBP 6.93 for Abasaglar.

#### 3.2.2. Northern Ireland, Scotland and Wales

The cost per PFD for Northern Ireland, Scotland, and Wales differed from England, as it was stable for Abasaglar from 2020 to 2024 but dropped for Lantus in these three countries. In 2022, the cost per Lantus pen was lower than the cost per Abasaglar pen, and this was sustained for 2023 and 2024 (Figure 5). As for England, the cost per generically prescribed iGlar closely matched the RP, Lantus. In Wales, the cost per PFD was generally closer to England, although, the rise seen in England in 2024 was not as steep in Wales. In Wales in 2024, the actual price per PFD for Lantus and Abasaglar was GBP 6.52 and GBP 6.62, respectively, which is considerably lower than the list price (Lantus GBP 6.95 and Abasaglar GBP 7.056 [12]).

In 2024, the cost of a PFD of Lantus and Abasaglar matched the list price, in line with prescribing data for Scotland. Note that costs in the prescribing data from Scotland are gross costs and not actual costs. As such, the findings here should be interpreted slightly differently, as they represent changes in list prices (iGlar was not prescribed generically in Scotland between 2020 and 2024).

### 3.3. Analysis of the Most Commonly Prescribed iGlar and Actual Cost per Cartridge and PFD

Here, we display our analysis of PCO prescribing of the least expensive (actual price, where available) iGlar. This analysis combined prescribing for cartridges with PFDs. Furthermore, it excluded Semglee as prescribing of this product was very low, reaching a maximum of 3.5% in England (2022), 0.5% in Scotland (2023), and 0.4% in Wales (2022). Semglee was not prescribed at all in Northern Ireland between 2020 and 2024.

**Figure 6:**
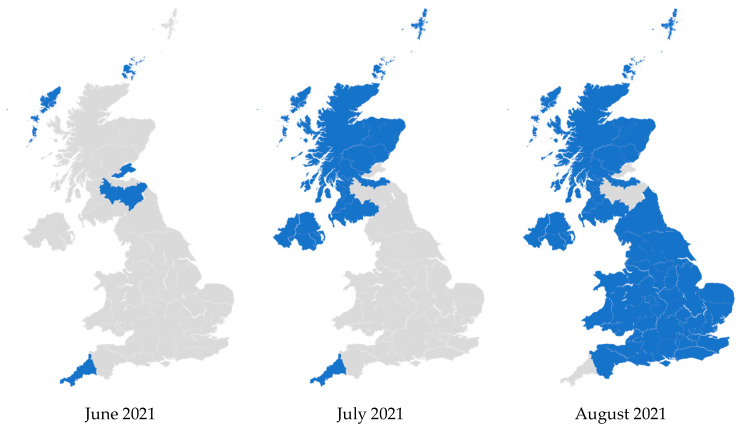
Regional variation of insulin glargine prescribing, 2020 to 2024. Figure legend: Blue: Primary care organizations where the most commonly prescribed insulin (in terms of prescribed quantity) was also the least expensive (actual price, where available). Grey: Primary care organizations where the most commonly prescribed insulin was not the least expensive.

In 2021, Lantus became the least expensive iGlar in the whole of the UK. This occurred from June to July 2021 in Northern Ireland and Scotland, and from July 2021 to August 2021 in England and Wales. Prior to this, Lantus was consistently dominant but not the least expensive. Subsequently, it continued to be the most common and also became the least expensive option (Figure 6). These patterns were retained until December 2024.

In Figure 6, some PCOs were prescribing more Abasaglar than Lantus before June 2021. In July 2021 and August 2021, they seem to flip to no longer prescribing the least expensive iGlar for most of their patients. These PCOs were mostly prescribing Abasaglar, and it was the least expensive product in June or July 2021. After Lantus became less expensive, Abasaglar was still the most commonly prescribed iGlar in those PCOs, but it was no longer the least expensive.

## 4. Discussion

Our analysis reports prescribing changes for iGlar products between 2020 and 2024, across England, Northern Ireland, Scotland, and Wales. Overall, the utilization of Lantus, the RP, declined from 2020 to 2024, however, biosimilar prescribing remained too low to surpass Lantus. By the end of the analysis period, it was also less expensive than Abasaglar across all nations, which is a departure from the common expectation that biosimilars are less expensive than the RP [2,3]. This is an important finding in our study, as it highlights the need for ongoing monitoring of available treatment options and their price. This means that policymakers in PCOs had the opportunity to make savings in July 2021 by switching back from Abasaglar to the RP, if they can work with prescribers to change prescribing behaviors that favored Abasaglar, although, this may be resource-intensive and lead to confusion for patients and healthcare practitioners.

The introduction of biosimilars had widely been anticipated to reduce the overall cost of biologic drugs as they are generally priced 10% to 30% less than the RP, though this has not been realized in all countries for other products [18]. The expectation from biosimilar manufacturers is that their products would be prioritized by payers. However, this was not the case with iGlar in the UK. The RP’s manufacturer reduced its list price after the launch of Abasaglar, and it maintained high prescribing levels. In addition to reductions in the list price, other discounts identified in our analysis have pushed the price even lower in England and Wales. This strategy appears to have worked in favor of most commissioners in the four nations as they obtained the least expensive product without implementing wide-scale change initiatives, which can be time-, labor-, and resource-intensive.

iGlar biosimilar adoption in the UK increased over the timeframe of our analysis, especially in England. However, a low price does not guarantee success, especially with commoditized therapies, as evidenced by Semglee remaining at low prescribing levels, suggesting that factors beyond price are significant. The uptake of Semglee may have been hindered by the severe shortages during the COVID-19 pandemic [19] and the product recall in 2022 [20]. In 2018, Aladul et al. reported that UK diabetology physicians had expressed dissatisfaction with the indication extrapolation of Lantus to Abasaglar [21]. Physicians also reported previous bad experiences with biosimilars, subsequently withdrawn from the market (e.g., Marvel Insulin [22]), and the lack of clarity over how cost savings are shared [21]. Other factors may include slow or absent formulary changes, entrenched prescriber behavior, difficulties in implementing change management among over-burdened prescribers, lack of prescriber awareness, ineffective patient education, and patient preference. Patient preference for originator brands has been documented in multiple studies in different therapy areas [23,24,25], though there is evidence to support erosion of this preference with generics becoming more common and more widely used [26]. In addition, the UK’s centralized procurement, clinical governance, and provider hospital Trust systems may have influenced biosimilar adoption.

The RP’s strong presence, coupled with its low price, makes the UK a challenging environment for future manufacturers of iGlar. This may discourage future iGlar biosimilar manufacturers from entering the market, which could have negative implications for commissioners in terms of price competition over the long term. In fact, prices may increase if biosimilars fail to find their place and are forced out of the UK.

Assuming a less expensive product always saves costs may be a mistake. With few peer-reviewed economic evaluations of biosimilars [18], decision-makers should consider the sustainability of supply, especially given recent medication shortages in the UK and globally [27].

To complement our quantitative findings, we suggest future research considers the qualitative drivers and barriers to prescribing for iGlar products. Specific questions may include why Semglee adoption was consistently low and why Lantus preference was maintained, which our analysis was not equipped to answer. Qualitative research could explore the attitudes of patients, pharmacists, prescribers, and payers.

## 5. Limitations

A cornerstone of the price per PFD analysis relied on the number of PFDs being prescribed correctly. However, it is entirely feasible that some prescriptions were issued with the total milliliters, milligrams, or units as the quantity, whereas others used the number of cartridges or PFD devices. We suspect the impact of this limitation to be low as data from Scotland, which utilized the gross ingredient cost was in line with current list prices. Additionally, the price per cartridge was the same as or very similar to the price per PFD for each brand. Last, we investigated variance in prescribing at the PCO level and found the standard deviation to be highest for Lantus in 2020 (GBP 0.23 for both cartridges and pens) and lowest for Semglee in 2021 (GBP 0.009). All of these factors taken together indicate the variance in price was limited and that any prescribing errors around the quantity are likely to be inconsequential.

Other errors in the prescribing data were not accounted for, neither was it possible to account for discounts not reported in the prescribing data that influence the final net price to payers due to the absence of data.

Variations in regional or PCO-level formularies and implementation of switching policies from biosimilar launch may have influenced prescribing trends. The exclusion of hospital prescribing may limit the generalizability of findings to the entire UK prescribing landscape. However, our analysis did show that hospital prescribing accounted for 2.8% of prescribing in England in 2024, suggesting that the impact of hospital prescribing is unlikely to change the final outcomes (detailed in the Supplementary Materials). The indication of treatment was not considered as changes in the prevalence or treatment approach may impact prescribing behavior, we assume the majority of prescribing was to treat patients with type 2 diabetes, which has a stable prevalence over 5 years. The impact of the COVID-19 pandemic was not investigated; however, long-acting insulin is a chronic medication, and changes in prescribing trends from acute infections are not thought to have a sustained impact.

## 6. Conclusions

Despite declining utilization, Lantus remains the most commonly prescribed iGlar in all countries over the 5-year analysis period. By 2024, Lantus was also priced lower than Abasaglar in every UK nation. This analysis indicates that the introduction of biosimilars does not automatically result in altered prescribing practices to favor a less expensive product.

Since Abasaglar was launched, several new therapies for type 2 diabetes have been made available to patients, including SGLT2 inhibitors and GLP-1 receptor agonists. This also applies to type 1 diabetes with long-acting insulin degludec and continuous insulin pumps that utilize rapid-acting insulin. This evolving landscape has reduced the role of iGlar, which may account for the long-term decline in its demand in the UK.

## Figures and Tables

**Table 1:** Insulin glargine products reported in the Dictionary of Medicines and Devices (January 2025) [12].

Virtual Medicinal Products (VMPs)	Actual Medicinal Products (AMPs)
Insulin glargine 100 units/mL solution for injection 3 ml cartridges BNF 0601012V0AAAAAA	Abasaglar 100 units/mL solution for injection 3 ml cartridges (Eli Lilly and Company Ltd.) BNF 0601012V0BDABAA
	Lantus 100 units/mL solution for injection 3 ml cartridges (Sanofi) BNF 0601012V0BBAAAA
Insulin glargine 100 units/mL solution for injection 3 ml pre-filled disposable devices BNF 0601012V0AAADAD	Abasaglar KwikPen 100 units/mL solution for injection 3 ml pre-filled pens (Eli Lilly and Company Ltd.) BNF 0601012V0BDACAD
	Lantus 100 units/mL solution for injection 3 ml pre-filled SoloStar pens (Sanofi) BNF 0601012V0BBAEAD
	Semglee 100 units/mL solution for injection 3 ml pre-filled pens (Biosimilar Collaborations Ireland Ltd.) BNF 0601012V0BEAAAD

BNF: British National Formulary. AMPs without a BNF code were not included. NHSBSA Copyright 2025 applies to BNF codes [13].

## Data Availability

All prescribing data are openly available from the NHS Business Service Authority Open Data Portal [14], Open Data NI [15], Public Health Scotland [16], and NHS Wales Shared Services Partnership [17]. Hospital prescribing data for England are also available from the NHS Business Service Authority [28].

## Supplementary Materials

The following supporting information can be downloaded at: https://www.mdpi.com/article/10.3390/pharmacy13030085/s1, Figure S1: Monthly quantity of branded prescribing for insulin glargine in England 2020 to 2024, inclusive; Figure S2: Monthly actual cost (GBP) of branded prescribing for insulin glargine in England 2020 to 2024, inclusive; Figure S3: Monthly quantity of branded prescribing for insulin glargine in Northern Ireland 2020 to 2024, inclusive; Figure S4: Monthly actual cost (GBP) of branded prescribing for insulin glargine in Northern Ireland 2020 to 2024, inclusive; Figure S5: Monthly quantity of branded prescribing for insulin glargine in Scotland 2020 to 2024, inclusive; Figure S6: Monthly gross ingredient cost (GBP) of branded prescribing for insulin glargine in Scotland 2020 to 2024, inclusive; Figure S7: Monthly quantity of branded prescribing for insulin glargine in Wales 2020 to 2024, inclusive; Figure S8: Monthly actual cost (GBP) of branded prescribing for insulin glargine in Wales 2020 to 2024, inclusive; Table S1: Insulin glargine products reported in the Dictionary of Medicines and Devices (January 2025); Table S2: Prescribing of iGlar 100 units/mL cartridges and pre-filled devices in hospitals in England in 2024; Table S3: Prescribing trends for iGlar 100 units/mL in England, 2020 to 2024; Table S4: Prescribing trends for iGlar 100 units/mL in Northern Ireland, 2020 to 2024; Table S5: Prescribing trends for iGlar 100 units/mL in Scotland, 2020 to 2024; Table S6: Prescribing trends for iGlar 100 units/mL in Wales, 2020 to 2024; Table S7: Quantity and cost per cartridge or pre-filled device PCO-level analysis.
