# Supplementary material for: Insulin Glargine Biosimilar Prescribing and Cost Trends in the United Kingdom’s Primary Care from 2020 to 2024
Source: Pharmacy (Basel). 2025 Jun 14;13(3):85. doi: 10.3390/pharmacy13030085 (PMC12196553; doi:10.3390/pharmacy13030085)
Supplement: Supplementary file 1 [file pharmacy-13-00085-s001.zip › pharmacy-3640346-supplementary.pdf]

pharmacy

MDPI

Research article – Supplementary Material for

Insulin Glargine Biosimilar Prescribing and Cost trends in the United Kingdom's Primary Care from 2020 to 2024

Table s1: Insulin glargine products reported in the Dictionary of Medicines and Devices (January 2025)

Virtual Therapeutic Moiety (VTM)	Virtual Medicinal Products (VMP)	Actual medicinal products (AMP)
Insulin glargine<br>VTM 776343005	Insulin glargine 100 units/ml solution for injection 10ml vials<br>VMP 36047111000001106<br>BNF 0601012V0AAABAB	Lantus 100 units/ml solution for injection 10ml vials (Sanofi)<br>AMP 3287911000001100<br>BNF 0601012V0BBABAB
	Insulin glargine 100 units/ml solution for injection 3ml cartridges<br>VMP 38896911000001108<br>BNF 0601012V0AAAAAA	Abasaglar 100 units/ml solution for injection 3ml cartridges (Eli Lilly and Company Ltd)<br>AMP 30172211000001102<br>BNF 0601012V0BDABAA
		Lantus 100 units/ml solution for injection 3ml cartridges (Sanofi)<br>AMP 3284211000001102<br>BNF 0601012V0BBAAAA
	Insulin glargine 100 units/ml solution for injection 3ml pre-filled disposable devices<br>VMP 38897011000001107<br>BNF 0601012V0AAADAD	Abasaglar KwikPen 100 units/ml solution for injection 3ml pre-filled pens (Eli Lilly and Company Ltd)<br>AMP 30171811000001105<br>BNF 0601012V0BDACAD
		Lantus 100 units/ml solution for injection 3ml pre-filled SoloStar pens (Sanofi)<br>AMP 11933011000001106<br>BNF 0601012V0BBAEAD
		Semglee 100 units/ml solution for injection 3ml pre-filled pens (Biosimilar Collaborations Ireland Ltd)<br>AMP 36082811000001107<br>BNF 0601012V0BEAAAD
	Insulin glargine 300 units/ml solution for injection 1.5ml pre-filled disposable devices<br>VMP 29903611000001103<br>BNF 0601012V0AAAEAE	Toujeo 300 units/ml solution for injection 1.5ml pre-filled SoloStar pens (Sanofi)<br>AMP 29866811000001104<br>BNF 0601012V0BCAAAE
	Insulin glargine 300 units/ml solution for injection 3ml pre-filled disposable devices<br>VMP 36931811000001104<br>BNF 0601012V0AAAFAF	Toujeo 300 units/ml solution for injection 3ml pre-filled DoubleStar pens (Sanofi)<br>AMP 36911311000001101<br>BNF code 0601012V0BCABAF

BNF: British National Formulary. AMPs without a BNF code were not included. NHSBSA Copyright 2025 applies to BNF codes [1].

Hospital-based prescribing of iGlar cartridges and pre-filled devices (PFDs) in England

To ensure our community-based focus was appropriate and representative of all prescribing in the UK, we investigated iGlar prescribing in hospitals in England. These data [2] are published every month at the Virtual Medicinal Product-(VMP) level, which is the generic description of the product. Hence, they do not distinguish between products from different manufacturers. Data were extracted in May 2025, covered the year from January 2024 to December 2024 and were based on the VMP codes denoted in Table s1.

All data were extracted and aggregated using R version 4.2.2, The R Foundation for Statistical Computing, and further analyzed with Microsoft Excel, Microsoft Office Professional Plus 2021.

For the year 2024, we found hospitals in England prescribed a total of 9,439 cartridges and 222,411 PFDs, or 2.8% of all iGlar products in scope of this study. A month-by-month breakdown is presented in Table s2.

Table s2: Prescribing of iGlar 100 units/mL cartridges and pre-filled devices in hospitals in England in 2024

	Cartridges	Pre-filled devices
January	917	19,086
February	805	16,911
March	881	17,985
April	744	18,496
May	776	18,759
June	804	17,407
July	699	18,714
August	616	18,539
September	702	17,154
October	657	20,010
November	899	18,767
December	940	20,583
**2024 total**	**9,439**	**222,411**

Community-based prescribing trends in England

Table s3: Prescribing trends for iGlar 100 units/mL in England, 2020 to 2024

	**Quantity**					**Actual cost** (GBP)				
	**2020**	**2021**	**2022**	**2023**	**2024**	**2020**	**2021**	**2022**	**2023**	**2024**
iGlar Cart	150,414	110,988**	87,577**	65,222**	49,829**	£1.1M	£756,807	£569,365	£425,506	£340,542
iGlar PFD	360,060	315,572*	241,545**	183,864**	139,588**	£2.5M	£2.2M	£1.6M	£1.2M	£955,007
Lantus Cart	1.1M	979,506**	918,214**	829,847**	760,537**	£7.8M	£6.7M	£6.0M	£5.4M	£5.2M
Lantus Pen	5.4M	5.1M*	5.1M	5.1M	5.3M*	£38.3M	£34.9M	£33.1M	£33.4M	£36.0M
Abasaglar Cart	81,484	77,894	75,292	75,387	78,544*	£534,567	£512,405	£496,960	£499,337	£543,046
Abasaglar Pen	980,608	1.1M*	1.2M**	1.3M**	1.5M**	£6.4M	£7.1M	£7.8M	£8.7M	£10.6M
Semglee Pen	91,226	177,493**	276,001**	261,067	256,188	£508,978	£993,157	£1.5M	£1.5M	£1.5M
**Total**	**8.2M**	**7.9M**	**7.9M**	**7.8M**	**8.1M**	**£57.1M**	**£53.1M**	**£51.0M**	**£51.1M**	**£55.1M**

Cart: cartridge, iGlar: insulin glargine, M: million, PFD: pre-filled device
In a paired, two tailed t-test * $p < 0.05$, or ** $p \leq 0.001$, vs. the previous year.

Figure s1: Monthly quantity of branded prescribing for insulin glargine in England 2020 to 2024, inclusive

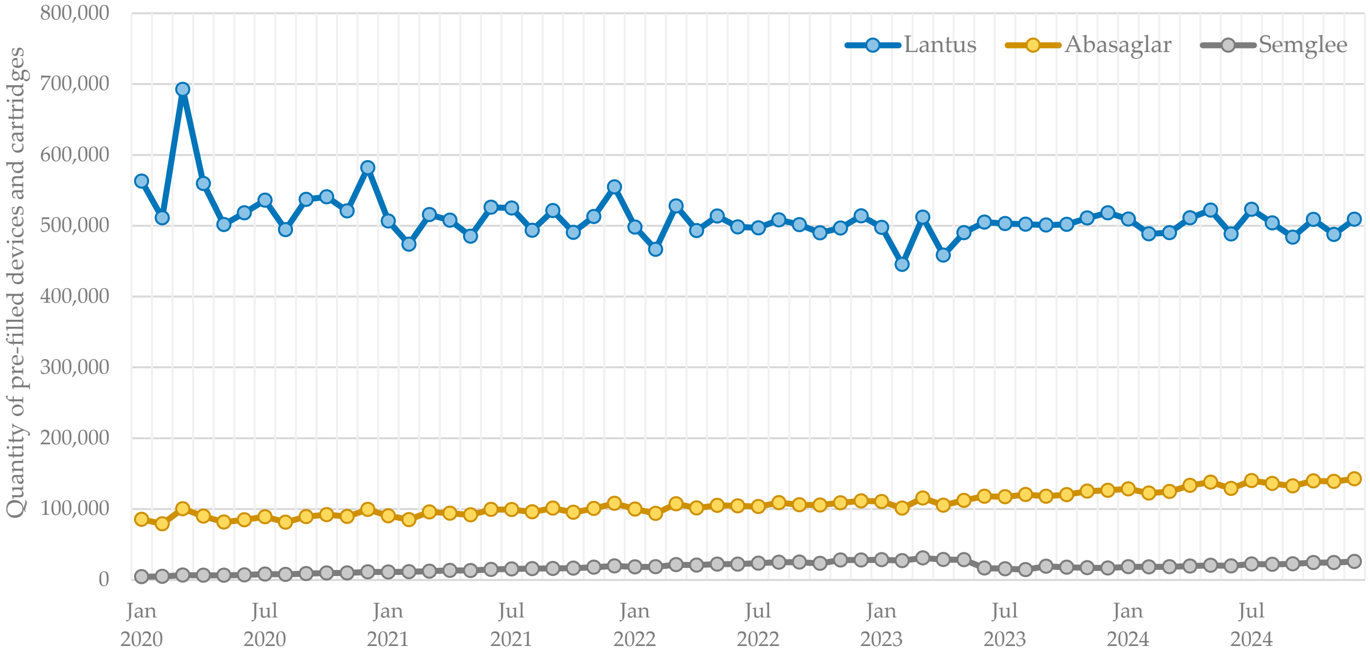

Figure s2: Monthly actual cost (GBP) of branded prescribing for insulin glargine in England 2020 to 2024, inclusive

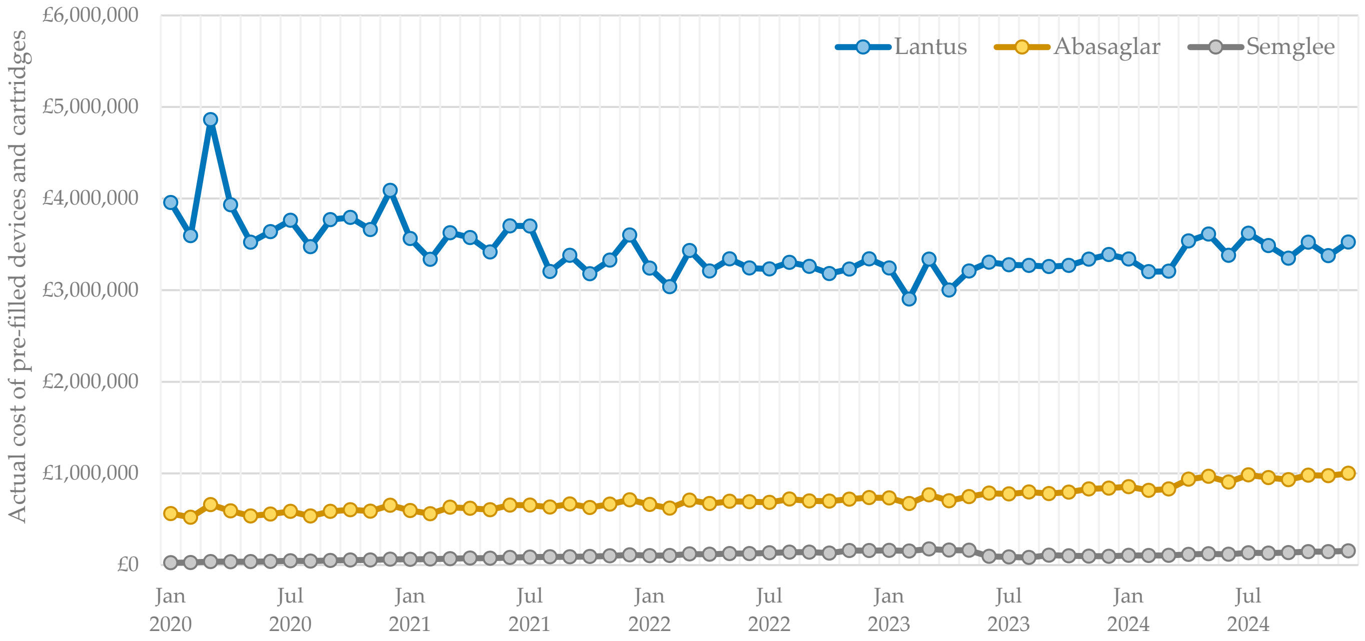

Community-based prescribing trends in Northern Ireland

Table s4: Prescribing trends for iGlar 100 units/mL in Northern Ireland, 2020 to 2024

	**Quantity**					**Actual cost** (GBP)				
	**2020**	**2021**	**2022**	**2023**	**2024**	**2020**	**2021**	**2022**	**2023**	**2024**
iGlar Cart	638	495	385	520	450	£4,819	£3,618	£2,676	£3,614	£3,128
iGlar PFD	2,622	1,900*	1,343	2,606*	2,551	£19,807	£13,918	£9,334	£18,112	£17,729
Lantus Cart	16,119	13,865*	13,528	11,922*	10,870	£121,763	£100,289	£94,020	£82,858	£75,547
Lantus Pen	280,510	264,621*	252,782*	235,023*	223,198	£2.1M	£1.9M	£1.8M	£1.6M	£1.6M
Abasaglar Cart	743	567	369	394	193	£5,243	£4,001	£2,604	£2,780	£1,362
Abasaglar Pen	11,657	12,379	12,778	12,323	12,180	£82,252	£87,346	£90,162	£86,951	£85,942
Semglee Pen	0	0	0	0	0	£0	£0	£0	£0	£0
**Total**	**312,289**	**293,827**	**281,185**	**262,788**	**249,442**	**£2.4M**	**£2.1M**	**£2.0M**	**£1.8M**	**£1.7M**

Cart: cartridge, iGlar: insulin glargine, M: million, PFD: pre-filled device
In a paired, two tailed t-test * p <0.05, or ** p≤0.001, vs. the previous year.

Figure s3: Monthly quantity of branded prescribing for insulin glargine in Northern Ireland 2020 to 2024, inclusive

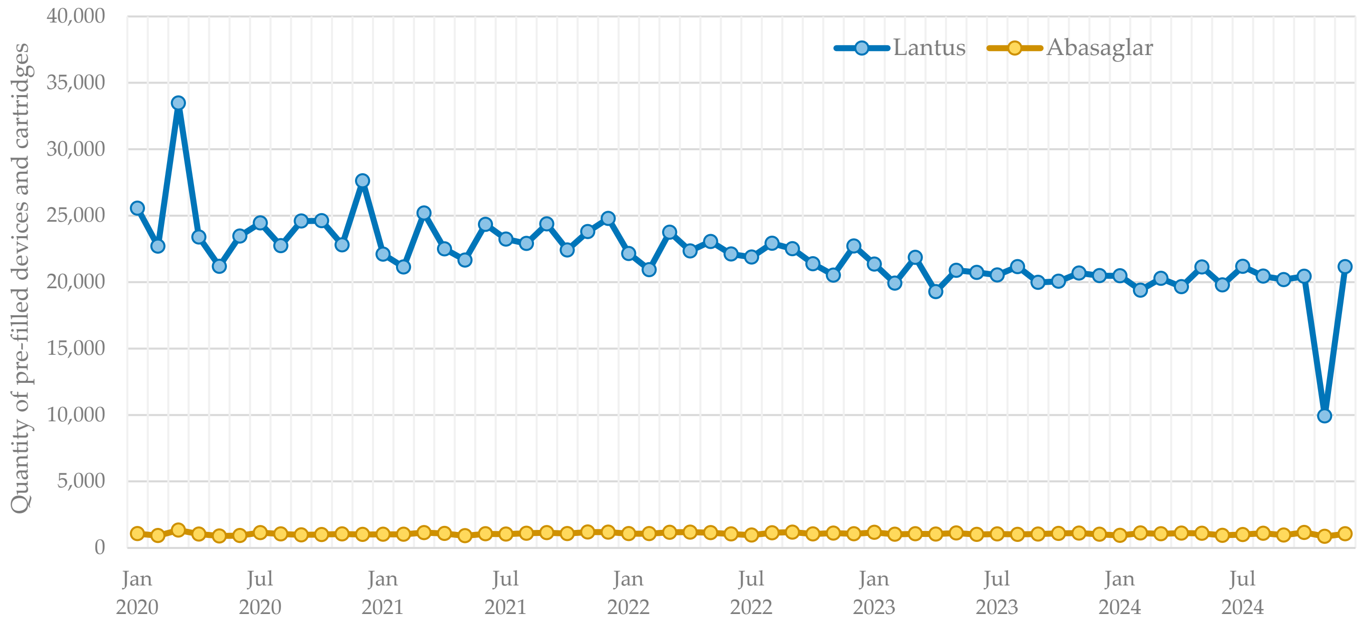

Figure s4: Monthly actual cost (GBP) of branded prescribing for insulin glargine in Northern Ireland 2020 to 2024, inclusive

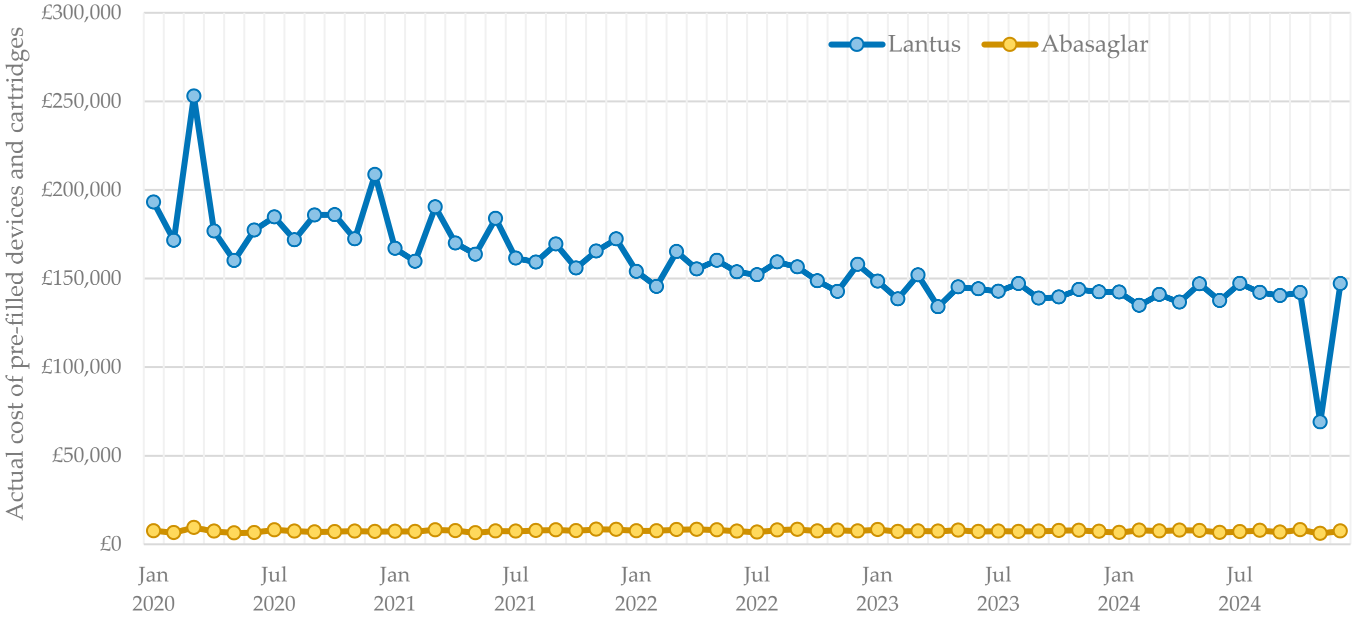

Community-based prescribing trends in Scotland

Table s5: Prescribing trends for iGlar 100 units/mL in Scotland, 2020 to 2024

	**Quantity**					**Actual cost** (GBP)				
	**2020**	**2021**	**2022**	**2023**	**2024**	**2020**	**2021**	**2022**	**2023**	**2024**
iGlar Cart	0	0	0	0	0	£0	£0	£0	£0	£0
iGlar PFD	0	0	0	0	0	£0	£0	£0	£0	£0
Lantus Cart	122,125	108,572*	100,203*	89,731*	81,750*	£922,532	£786,414	£696,318	£623,603	£568,163
Lantus Pen	437,200	411,916*	398,685*	381,824*	386,196	£3.3M	£3.0M	£2.8M	£2.7M	£2.7M
Abasaglar Cart	8,640	7,697*	7,253	6,485	6,062	£60,964	£54,310	£51,177	£45,758	£42,773
Abasaglar Pen	131,663	132,999	134,905	135,391	136,903	£928,986	£938,385	£951,841	£955,298	£965,988
Semglee Pen	0	0	0	2,889**	823*	£0	£0	£0	£17,328	£4,936
**Total**	**699,628**	**661,184**	**641,046**	**616,320**	**611,734**	**£5.2M**	**£4.8M**	**£4.5M**	**£4.3M**	**£4.3M**

Cart: cartridge, iGlar: insulin glargine, M: million, PFD: pre-filled device
In a paired, two tailed t-test * p <0.05, or ** p≤0.001, vs. the previous year.

Figure s5: Monthly quantity of branded prescribing for insulin glargine in Scotland 2020 to 2024, inclusive

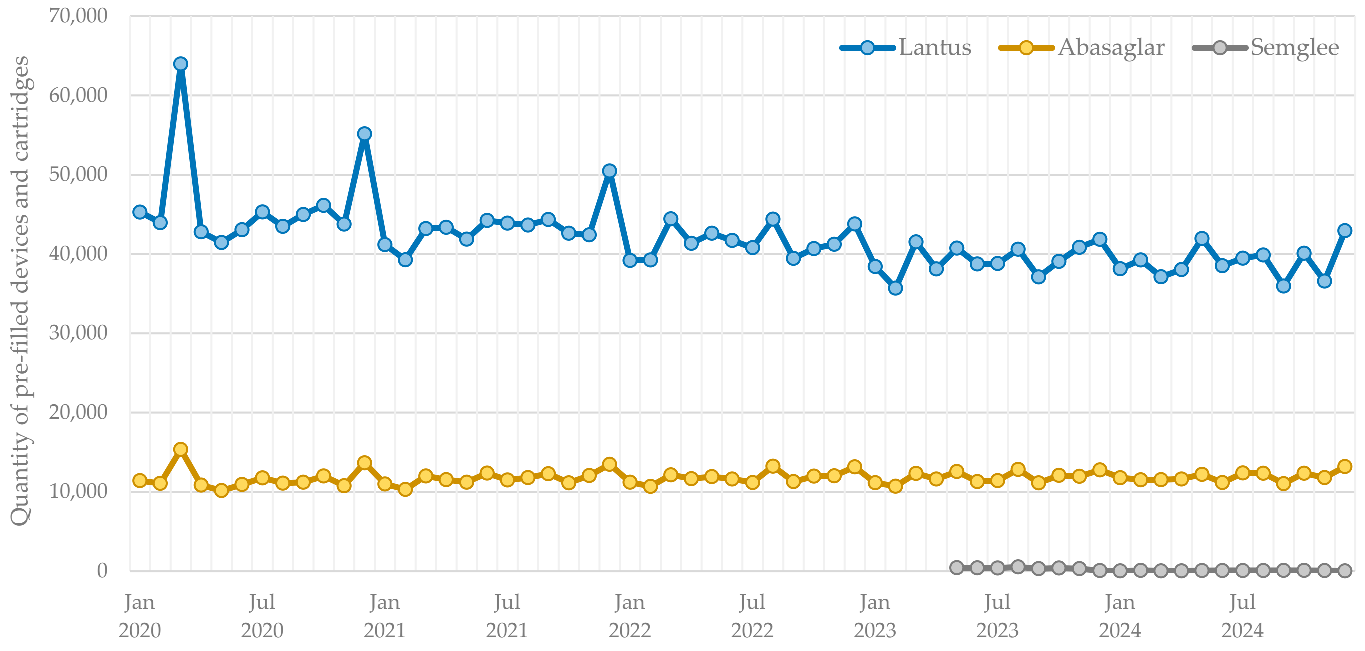

Figure s6: Monthly gross ingredient cost (GBP) of branded prescribing for insulin glargine in Scotland 2020 to 2024, inclusive

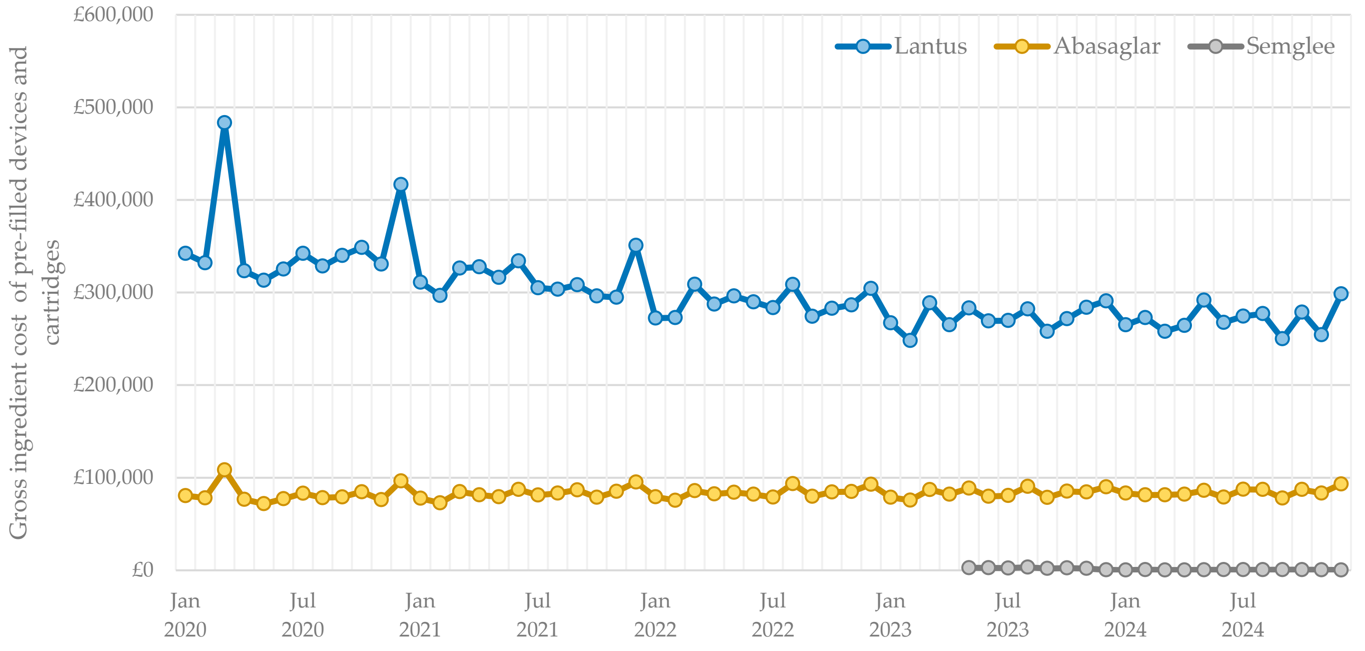

Community-based prescribing trends in Wales

Table s6: Prescribing trends for iGlar 100 units/mL in Wales, 2020 to 2024

	Quantity					Actual cost (GBP)				
	2020	2021	2022	2023	2024	2020	2021	2022	2023	2024
iGlar Cart	3,936	2,990	2,295	1,720	1,263	£27,503	£20,257	£14,858	£11,141	£8,233
iGlar PFD	12,871	11,312*	9,582	7,476	6,188	£89,964	£76,653	£62,054	£48,437	£40,355
Lantus Cart	61,378	56,245**	50,631*	44,435*	39,396*	£428,929	£380,545	£327,808	£287,844	£256,878
Lantus Pen	514,351	474,429*	459,677*	443,885*	431,553	£3.6M	£3.2M	£3.0M	£2.9M	£2.8M
Abasaglar Cart	2,884	2,490	2,342	2,380*	2,279	£18,828	£16,293	£15,395	£15,652	£15,090
Abasaglar Pen	40,791	42,340	46,434	48,807	56,748	£266,333	£277,101	£305,264	£321,059	£375,769
Semglee Pen	926	1,244	2,009	1,549**	1,281*	£5,140	£6,924	£11,228	£8,664	£7,210
**Total**	637,137	591,050	572,970	550,252	538,708	£4.4M	£4.0M	£3.7M	£3.6M	£3.5M

Cart: cartridge, iGlar: insulin glargine, M: million, PFD: pre-filled device
In a paired, two tailed t-test * $p < 0.05$, or ** $p \leq 0.001$, vs. the previous year.

Figure s7: Monthly quantity of branded prescribing for insulin glargine in Wales 2020 to 2024, inclusive

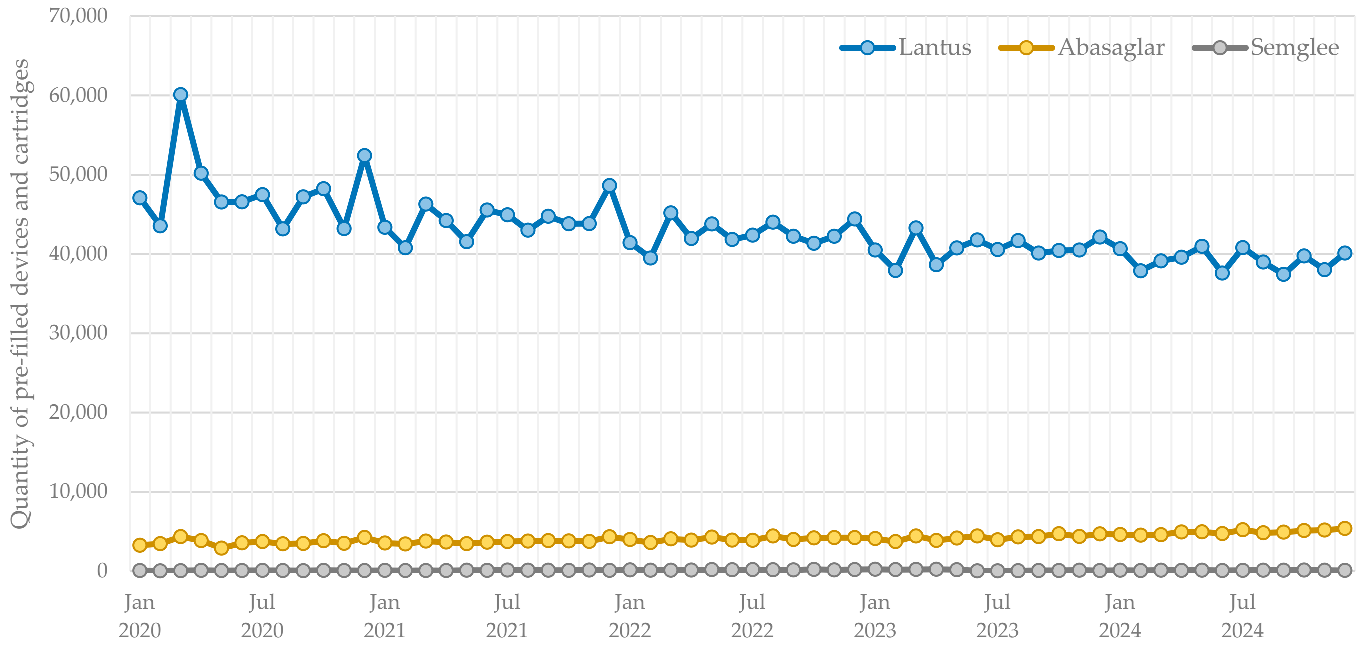

Figure s8: Monthly actual cost (GBP) of branded prescribing for insulin glargine in Wales 2020 to 2024, inclusive

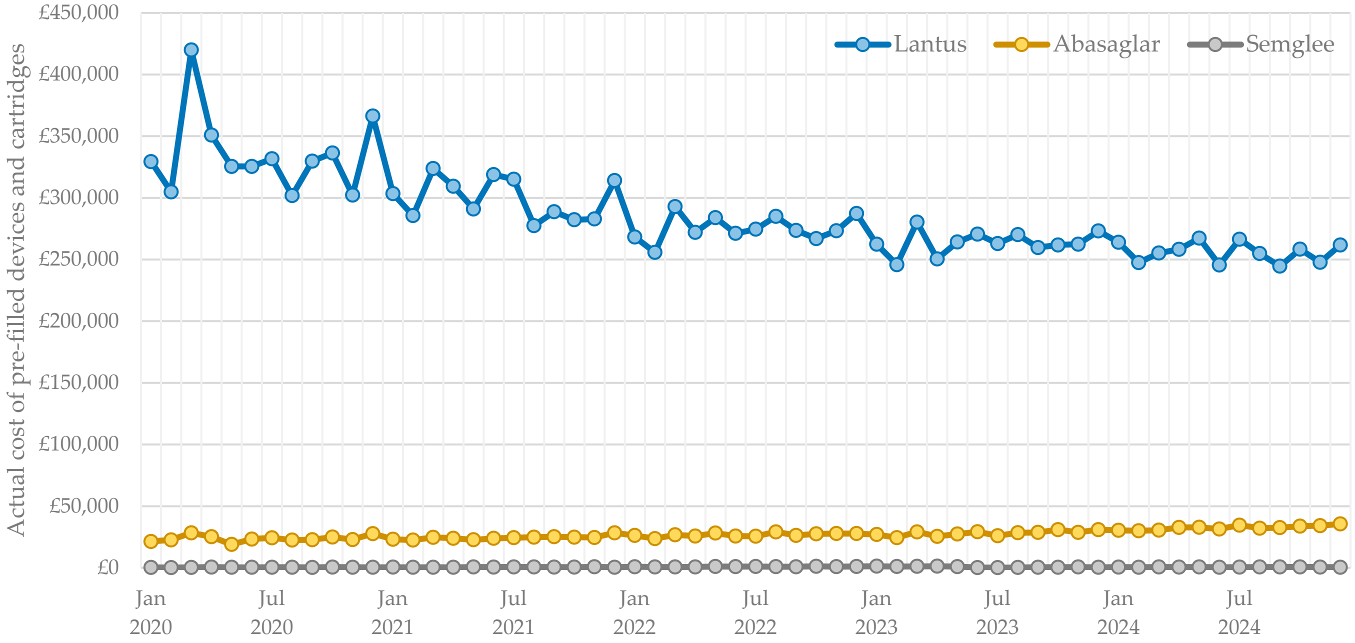

Variance in the quantity and cost per cartridge or PFD at the PCO level in the UK

To quantify the variance in prescribing cost per cartridge or PFD, we aggregated data to the PCO level and reviewed annual changes in the quantity and cost per cartridge or PFD as the mean, standard deviation, median and range (Table s7). At the PCO level, this analysis confirms the country-level analysis and shows both the average and median prices declining over time, in line with the country-level analyses.

Interestingly, the standard deviation consistently becomes smaller over time for all products, indicating reduced variability in the price per cartridge or PFD towards 2024. The cost per cartridge or PFD also shifts to a less expensive window over time for all products.

Table s7: Quantity and cost per cartridge or pre-filled device PCO-level analysis

		Quantity					Cost (GBP) per cartridge or pre-filled device				
**Product**	**Year**	**Mean**	**Std dev**	**Median**	**Max**	**Min†**	**Mean**	**Std dev**	**Median**	**Max**	**Min**
Lantus cartridge	2020	15,941	13,786	11,298	71,371	785	£7.14	£0.23	£7.02	£7.55	£6.99
	2021	14,124**	13,807	11,050	63,093	550	£6.92	£0.20	£6.81	£7.27	£6.76
	2022	13,201**	12,858	9,787	57,245	455	£6.62	£0.20	£6.50	£6.95	£6.47
	2023	11,901**	11,608	8,748	49,990	450	£6.64	£0.19	£6.52	£6.95	£6.48
	2024	10,884**	10,591	8,226	43,736	380	£6.82	£0.12	£6.83	£6.95	£6.52
Lantus Pen	2020	81,478	68,245	65,309	297,290	4,495	£7.14	£0.23	£7.02	£7.55	£6.99
	2021	76,643*	70,311	65,349	287,827	4,597	£6.92	£0.20	£6.81	£7.25	£6.77
	2022	75,576	69,536	62,518	276,679	3,856	£6.62	£0.20	£6.50	£6.95	£6.47
	2023	75,314	70,061	63,297	272,117	2,434	£6.64	£0.19	£6.53	£6.95	£6.48
	2024	76,901*	72,733	61,540	273,838	2,436	£6.83	£0.12	£6.84	£6.95	£6.52
Abasaglar cartridge	2020	1,143	1,457	591	7,688	10	£6.66	£0.20	£6.56	£7.06	£6.53
	2021	1,081	1,589	387	8,761	20	£6.69	£0.20	£6.58	£7.06	£6.54
	2022	1,040	1,521	418	8,270	25	£6.71	£0.20	£6.60	£7.06	£6.57
	2023	1,032	1,525	419	7,996	10	£6.73	£0.19	£6.62	£7.06	£6.58
	2024	1,062	1,578	414	8,141	12	£6.92	£0.13	£6.94	£7.06	£6.62
Abasaglar Pen	2020	14,204	15,282	7,825	79,405	15	£6.66	£0.20	£6.56	£7.06	£6.53
	2021	15,448*	18,193	7,384	84,934	5	£6.70	£0.21	£6.58	£7.06	£6.54
	2022	16,773**	19,949	8,192	91,853	25	£6.72	£0.20	£6.60	£7.06	£6.57
	2023	18,435**	21,973	8,572	99,712	25	£6.73	£0.19	£6.63	£7.06	£6.58
	2024	21,136**	25,404	9,922	119,787	15	£6.93	£0.12	£6.95	£7.06	£6.62
Semglee Pen	2020	1,124	2,571	65	15,724	10	£5.58	£0.01	£5.58	£5.59	£5.55
	2021	2,180**	5,016	93	30,107	20	£5.59	£0.01	£5.59	£5.61	£5.56
	2022	3,390**	7,748	172	45,458	25	£5.61	£0.01	£5.61	£5.64	£5.58
	2023	3,238	7,386	292	45,310	10	£5.71	£0.15	£5.63	£6.00	£5.59
	2024	3,150	7,331	153	43,265	5	£5.88	£0.10	£5.91	£6.00	£5.63

Max: maximum, Min: minimum, PCO: primary care organization, Std dev: standard deviation

In a paired, two tailed t-test * $p < 0.05$, or ** $p \leq 0.001$, vs. the previous year.

†Ignores PCOs that did not prescribe the product at all.

References

1. NHS Business Services Authority. BNF Code Information – Current Available online: https://opendata.nhsbsa.net/dataset/bnf-code-information-current-year (accessed on 2 May 2025).
2. NHS Business Services Authority. Provisional Secondary Care Medicines Data (SCMD) with Indicative Price Available online: https://opendata.nhsbsa.net/dataset/secondary-care-medicines-data-indicative-price (accessed on 16 May 2025).
